# Speed versus damage: using selective feedback to modulate laparoscopic simulator performance

**DOI:** 10.1186/s12909-021-02789-3

**Published:** 2021-06-29

**Authors:** Bas Kengen, Wouter M. IJgosse, Harry van Goor, Jan-Maarten Luursema

**Affiliations:** 1 Department of Surgery, Radboud University Medical Center, Geert Grooteplein zuid 10, Nijmegen, 6525 GA Gelderland The Netherlands; 2 PO Box 9101 (960), Nijmegen, 6500 HB The Netherlands

**Keywords:** Simulation, Laparoscopy, Skills development, Adaptive training, Personality

## Abstract

**Background:**

Adaptive training is an approach in which training variables change with the needs and traits of individual trainees. It has potential to mitigate the effect of personality traits such as impulsiveness on surgical performance. Selective performance feedback is one way to implement adaptive training. This paper investigates whether selective feedback can direct performance of trainees of either high- or low impulsiveness.

**Methods:**

A total of 83 inexperienced medical students of known impulsiveness performed a four-session laparoscopic training course on a Virtual Reality Simulator. They performed two identical series of tasks every session. During one series of tasks they received performance feedback on duration and during the other series they received feedback on damage. Performance parameters (duration and damage) were compared between the two series of tasks to assess whether selective performance feedback can be used to steer emphasis in performance. To assess the effectiveness of selective feedback for people of high- or low impulsiveness, the difference in performance between the two series for both duration and damage was also assessed.

**Results:**

Participants were faster when given performance feedback for speed for all exercises in all sessions (average z-value = − 4.14, all *p* values < .05). Also, they performed better on damage control when given performance feedback for damage in all tasks and during all sessions except for one (average z-value = − 4.19, all but one *p* value < .05). Impulsiveness did not impact the effectiveness of selective feedback.

**Conclusion:**

Selective feedback on either duration or damage can be used to improve performance for the variable that the trainee receives feedback on. Trainee impulsiveness did not modulate this effect. Selective feedback can be used to steer training focus in adaptive training systems and can mitigate the negative effects of impulsiveness on damage control.

## Background

Personality is a major source of differences in behavior between people [1–3]. Emerging research is highlighting differences in personality between surgeons and controls; in these studies surgeons typically show heightened extraversion [4–6]. In traffic and in high-skilled professions such as pilots, the related personality trait of impulsiveness has been shown to correlate with dangerous behavior [7–12]. Patients may be at risk if a similar association is present in the operating room. In a previous simulator-based laparoscopic training study, we found that high-impulsiveness trainees created more damage in comparison to low-impulsiveness trainees but were equally fast [4]. An adaptive training approach, already used in military medical skills acquisition and retention, to effectively train personnel of different skills levels [13], could potentially counteract the negative effects associated with high impulsiveness.

In adaptive training, variables such as the difficulty level of the training task are varied as a function of trainee performance, to maximize learning and keep the trainee’s interest level high [14]. Many different forms of adaptive training have been described [15]. In its simplest form it means adapting the difficulty of the exercise based on the performance of the apprentice. Other examples are adjusting task difficulty to individual differences such as personality or learning styles, or altering perceived difficulty levels by modifying performance standards [16, 17]. Advantages of this type of training are among others: a personalized learning experience, focused remediation of individual weaknesses in skilled performance, and its ability to give teachers a better insight in the students’ capabilities. Adaptive training has been proven effective in a variety of novel educational fields [18–22], including virtual reality (VR) based training and serious gaming [23, 24].

VR training is increasingly used for the acquisition of psychomotor skills needed for minimally invasive surgery. One of the advantages of these electronic simulators is the large amount of quantified performance parameters they record. Currently, this information is mostly used to provide feedback to its users to demonstrate their progress. However, this feature provides an opportunity to steer emphasis of a user to a specific aspect of a task, for example speed or errors made. In this way performance parameters could be used to create a form of adaptive training. Such personalized training which steers the student towards improving his or her weaknesses may increase training quality and efficiency. A previous review indicated that different skills benefit from different types of feedback, for example process feedback may be a more effective way to train decision making than outcome feedback [15]. However, little is known about types of feedback in relation to surgical skills training.

The research reported here investigated two questions: Can selective feedback be used to steer student performance towards either damage control or speed in laparoscopic simulator training? If so, does impulsiveness impact these changes? We expected selective feedback to influence performance positively for the targeted performance measure. We did not formulate an expectation as to whether the effect of selective feedback would be impacted by impulsiveness.

## Materials and methods

### Subjects and study design

Every month, around thirty first-year master students of Medicine with no- or very limited surgical experience start their surgical rotations at the Radboud University Medical Center. These students were given the opportunity to voluntarily sign up for a basic laparoscopic skills simulation training course as part of their rotation. During all training sessions, students performed two series of exercises that only differed in whether feedback was provided on speed or on damage control. We collected data over a period of 6 months for a total of 83 participants. Students were explicitly told that enrollment in the study was voluntary and declining would not impact their participation to the course or the assessment of their rotation. All students elected to participate, and filled out a digital demographics- and impulsiveness questionnaire. Performance on time and damage was compared for both feedback series, and within each series for students of high- and low impulsiveness. The study design was not submitted to an ethical board, as this was not required for this type of research under Dutch law at the time of data collection [25]. Voluntary informed consent was obtained from all participating students.

### Course design

**Fig. 1:**
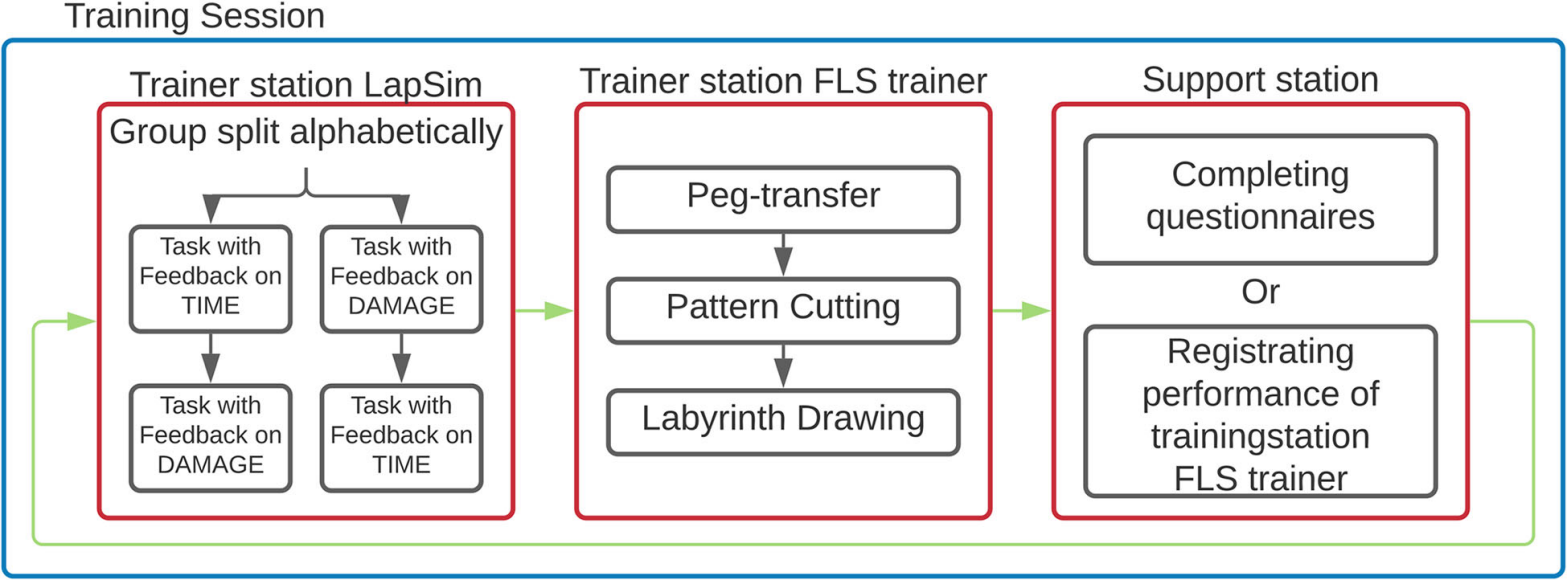
Flowchart for a single training session. Participants always rotated between the stations in the same order and performed at each station once in each session. The total course consisted of four of these training sessions

The course consisted of four 60-min sessions, scheduled on different days to maximize learning [26, 27]. The four training sessions were performed within 3 weeks, with no more than one training session scheduled on a single day (distributed practice). Previous research demonstrated similar retention of a complex surgical motor skill for a weekly and a monthly training schedule [28]. We do not expect different time intervals between sessions to result in significant performance differences. Self-selected groups of three students scheduled their sessions in an online calendar. Participating students were assigned a random login code to the VR simulator to ensure anonymity. During each session, students would rotate along the VR trainer station, the Fundamentals of Laparoscopic Surgery (FLS) trainer station, and a support station to assist the student at the FLS trainer station with collecting performance data (which was not automated for this station as it was for the VR station) (Fig. 1). During the first session the students were introduced to the available training stations by one of the researchers. The other training sessions were not supervised. The participants started at the same training station every session.

During the first session students completed two digital questionnaires while they were at the support station: a digital demographics questionnaire including questions for previous laparoscopic (simulator) experience, and a digital version of the Eysenck Impulsiveness Inventory to collect information about impulsiveness.

### Training stations: the LapSim VR trainer and the FLS videotrainer

#### LapSim VR trainer station

At this station, during each session, students performed two series of the same four tasks on the LapSim VR trainer. The LapSim Virtual Reality trainer is a well-researched simulator and transfer of skills gained from training on the LapSim to operating room performance has been established [29–31]. The series differed only in feedback emphasis: during one series, students received feedback via the simulator on duration only, and for the other they received feedback via the simulator only on damage parameters. Limited feedback for both damage and duration was given during the tasks. The screen glowed red when participants inflicted virtual damage, and in one of the tasks, subtasks would end if the participant acted too slow. Quantitative summary feedback in relation to normative expert values was given at the end of each task for either speed or damage, implementing our experimental conditions. This consisted of time in seconds for duration (time on task), number of damage inflicting incidents (tissue damage), and deepest virtual wound in millimeters (maximum damage). Students were split alphabetically by their last name into two equal-sized groups to start with either the speed series or damage-control series. Both series were completed during each session by all students. Students were informed of this design, and knew when they were training for speed or for damage control. The four tasks were ‘camera navigation’, ‘instrument navigation’, ‘cutting’ and ‘lifting and grasping’. These are tasks where the student operates the camera or uses instruments such as a grasper or a ligation device in a simulated abdominal cavity to complete simple, non-procedural exercises involving simulated blood vessels, gall stones and suturing needles. Detailed descriptions of the tasks can be found at the website of Surgical Science [32].

#### FLS videotrainer station

To prepare students for their surgical rotation they also trained on the FLS trainer, but this data was not used for this research. On this videobox trainer students trained basic skills. Performed exercises were ‘peg-transfer’, ‘pattern cutting’ and ‘labyrinth drawing’. A description of the first two tasks can be found at the website of Fundamentals of Laparoscopic Surgery [33]. The last exercise is a self-developed task where students have to trace a path through a labyrinth using a marker attached to a laparoscopic instrument, designed to learn to anticipate the amplification of movement caused by working over a fulcrum with the laparoscopic instruments. The third student who was not training at a training station recorded the performance of the student practicing at the FLS videotrainer station to help them monitor progress.

### Simulators and apparatus

The LapSim VR trainer station consisted of a desktop computer connected to Simball Hardware (G-coder Systems, Västra Frölunda, Sweden), running Surgical Science’s LapSim® Virtual Reality Simulator training software v.3.0 (Göteborg, Sweden). This is a validated VR simulator designed to teach basic skills and some laparoscopic procedures [29, 34, 35]. Data was saved and stored in Microsoft Excel 2007 and analyzed with IBM SPSS Statistics for Windows, Version 22.0 (Armonk, NY: IBM Corp).

The FLS videotrainer is a validated box trainer developed by SAGES and ACS [36–38]. This box trainer was connected to a 17-in. Philips LCD monitor.

A laptop running windows 7 was installed for the students to complete the questionnaires. Questionnaires were created with Limesurvey Version 1.92+, a web application to create surveys.

The Eysenck Impulsiveness Inventory consists of 63 yes-no questions and was developed in 1978 by S. B. G. Eysenck and H.J. Eysenck for the measurement of three primary personality traits; impulsiveness, venturesomeness, and empathy [39–41]. Examples of these questions are “Do you often buy things on impulse?”, “Do you generally do and say things without stopping to think?” and “Do you often change your interests?”. Previous studies demonstrated good scale reliability for impulsiveness for this broadly used test, with a Cronbachs alpha ranging between 0.82–0.84 for impulsiveness [40, 42]. Reliability for venturesomeness and empathy demonstrated questionable to good reliability, with Cronbachs alpha ranging between 0.65–0.85.

### Data preparation/ analysis

Data on the LapSim was automatically registered by the simulator. The parameters instrument time and tissue damage were included in data-analysis, as these are the primary outcome measures of this research. Instrument time records the total duration of an exercise, tissue damage records the number of instances virtual damage was created. The task ‘Camera navigation’ was used as warming-up exercise and not analyzed. A *p* value of < .05 was considered significant.

Shapiro-Wilk tests demonstrated that not all of the data followed a normal distribution. For damage this was caused by a floor effect, as participants did not always created damage, which happened most often during the last training session. For time it was caused by a ceiling effect for the exercise ‘instrument navigation’, as there was a maximum time-limit which was reached by 21.3% of the students during the first session. During the remaining sessions this limit was reached by less than 2% of the students. Wilcoxon signed ranks tests were performed to compare the two training series. This was done for every exercise and session separately.

Two participants had limited laparoscopic camera assistant experience. Their performance however was between the first and third quartile for both damage control and speed, and their performance data was kept in the dataset. The other participants reported no laparoscopic experience, ensuring equal experience levels.

The Eysenck Impulsiveness Inventory measures impulsiveness, venturesomeness, and empathy. Impulsiveness has been shown to correlate with dangerous behavior in traffic, aviation and decision making [7–12]. As far as we know, for empathy and venturesomeness such links have not been demonstrated. Additionally, the locus of this study was a single-user laparoscopic basic skills course with simple, predictable exercises. In contrast to the more socially and technically complex environment of the operating room, we did not expect empathy or venturesomeness to affect training outcomes. To not negatively impact the power of our study by introducing additional variables, we focused on the personality trait of impulsiveness in this study. Impulsiveness scores were calculated at the end of the data-collection phase to prevent information bias for both students and researchers.

Based on the results of the Eysenck Impulsiveness Inventory the students were divided into two experimental groups after data collection, a group of low impulsiveness and a group of high impulsiveness. The low-impulsiveness group consisted of all the students that scored equal or lower than the median score, the high-impulsiveness group of all students that scored higher. There were no significant differences in the distribution of the impulsiveness-groups between the starting order of the training stations or training series.

To examine if students of high-impulsiveness and low-impulsiveness are equally suitable for this type of adaptive training, we compared the effect of altered feedback between the two groups. To do this, we subtracted performance parameters of the training series with emphasis on damage control from the performance parameters of the training series with emphasis on speed. The resulting differences were than compared between the two impulsiveness-groups with Mann-Whitney U tests. This was done for both speed and damage, for every exercise and session separately.

## Results

### Participants

A total of 83 students participated (Table 1). Of these students 62.7% completed all four sessions. Due to technical issues, data for two students was lost. Data of the remaining 81 participants were analyzed. Age ranged between 21 and 30 years (mean 23.6 years) and 26 participants were male (32.1%). The preferred hand was the right hand for 74 participants (91.4%). The groups of high- and low impulsiveness students did not differ for age, sex, preferred hand, and laparoscopic experience.

**Table 1:** Summary of characteristics of study participants

Group	Total	Low-impulsive	High-impulsive
**Number**	*n* = 81	*n* = 41	*n* = 40
**Sex**	26/81 male (32.1%)	12/41 male (29.2%)	14/40 male (35.0%)
**Age**	23.9 years (21–30 years)	23.3 years (21–30 years)	23.8 years (21–29 years)
**Preferred hand**	74/81 right hand (91.4%)	37/41 right hand (90.2%)	37/40 right hand (92.5%)
**Laparoscopic experience**	2/81 (2.5%)	1/41 (2.4%)	1/40 (2.5%)

### Differences in performance between the two training series

**Fig. 2:**
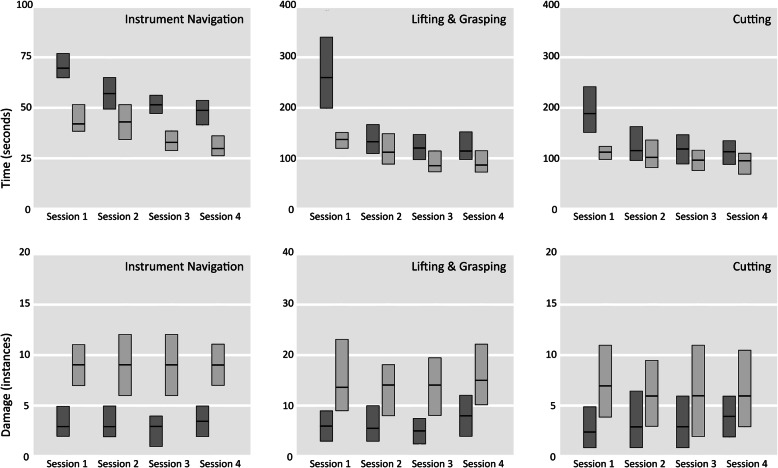
Performance comparison for the speed and damage series, for each session and task. Damage series performance is dark gray, speed series performance is medium gray. The black horizontal stripes indicate median values, the boxes indicate quartiles. All damage series and speeds series pairs are different, with the exception of damage in the first ‘Lifting & Grasping’ session

Comparisons for performance on speed and on damage, within both the speed and damage feedback series, are shown in Fig. 2. Participants were significantly faster when given performance feedback for speed for all exercises in all sessions; Participants performed also significantly safer when given performance feedback for damage, with the exception of the ‘Lifting & Grasping’ exercise during the first session (Table 2).

**Table 2:** Results of the Wilcoxon signed ranks tests comparing performance of every task between the two training series; speed versus damage control

	**Instrument navigation**							
	Total time				Tissue damage			
	session 1	session 2	session 3	session 4	session 1	session 2	session 3	session 4
Z-value	−4,20	−6,03	−6,57	−5,70	−3,71	−5,50	− 6,27	− 4,97
Significance	,00	,00	,00	,00	,00	,00	,00	,00
	**Lifting & Grasping**							
	Total time				Tissue damage			
	session 1	session 2	session 3	session 4	session 1	session 2	session 3	session 4
Z-value	−3,52	−2,81	−4,01	−4,30	−1,58	−4,59	−5,38	−4,39
Significance	,00	,01	,00	,00	,11	,00	,00	,00
	**Cutting**							
	Total time				Tissue damage			
	session 1	session 2	session 3	session 4	session 1	session 2	session 3	session 4
Z-value	−3,73	−3,57	−2,50	−2,72	−2,14	−3,90	−4,03	− 3,82
Significance	,00	,00	,01	,01	,03	,00	,00	,00

### Influence impulsiveness on performance differences between the two training series

**Fig. 3:**
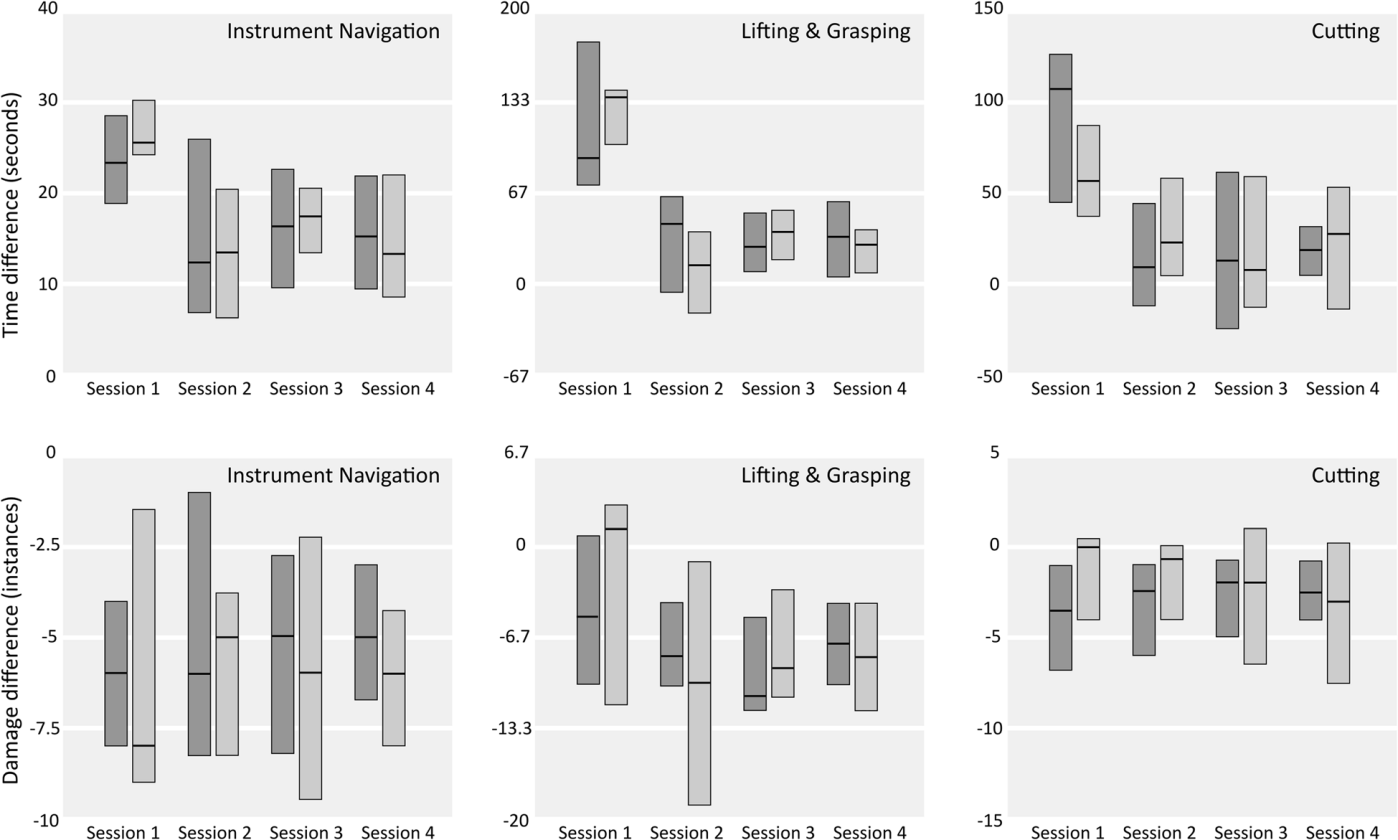
Performance differences between the damage and speed feedback series, compared for students of high and low impulsiveness. Low impulsiveness shown as dark gray, high impulsiveness as medium gray. Black horizontal stripes indicate median values, the boxes indicate quartiles. Feedback for damage and for speed induced the same performance differences for students of low- and high impulsiveness in all sessions of all tasks (and thus was equally effective for both groups)

Differences in performance between the two training series did not differ between the low and high-impulsiveness group for any task or session (Fig. 3). Even when comparing the first quartile of students of low-impulsiveness to the fourth quartile of students of high-impulsiveness no differences were found.

## Discussion

In adaptive training, task variables and task complexity change throughout the training experience to provide the trainee with an optimal challenge. Adaptive training optimizes training effectiveness and efficiency as it can accommodate individual differences between trainees in their path towards competency. Adaptive training systems have been proven effective in areas such as improving memory, rehabilitation, and x-ray screening [18–22]. There are several ways to implement adaptive training; for example gaming related levels that increase in difficulty based on the player’ skills level (seen in serious games [24, 43, 44]), or individual trajectories that steer trainees toward tasks designed to remedy specific lapses in skills or knowledge. In this study, we have established the potential use of selective performance feedback to implement adaptive training for surgical skills.

In earlier research we established the effect of impulsiveness on laparoscopic simulator performance [4]. High-impulsiveness students created more damage but were not faster in various basic skills tasks. As damage control is a major goal of surgical skills training, adaptive training could optimize training efficiency by emphasizing damage control related feedback for students of high impulsiveness. This could be straightforward to implement, as we found in this study that trainee performance was strongly biased towards either speed or damage control by the type of feedback they received, regardless of trainee impulsiveness status.

Finding that impulsiveness does not impact the effect of selective feedback contrasts with earlier research that found different personalities react differently to adaptive training, with personality traits such as openness to experience and neuroticism correlating positively with adaptive training outcomes [45]. Personality is a multi-faceted construct, as is surgical performance, and more studies are needed understanding the relations between this source of individual differences and surgical performance. Of special interest would be to study the relation between personality and operating room performance, where team functioning is an additional variable likely to be affected by personality. This relation would remain undetected during simulator skills training, which typically happens on an individual basis.

### Limitations

Our study has a few limitations. The task ‘instrument navigation’ has a time limit and shuts down if the items of the task are not completed before the limit is reached. During the first training session only, 21.3% of the students were not able to finish this task in the allotted time span. As a consequence, performance differences for this task during this session are smaller than they would have been under unlimited temporal conditions. Despite this limitation we found large and significant differences in performance on this task and session under selective feedback.

Also, we did not exclude students who did not complete all four sessions and this could potentially be a source of bias. Incomplete courses were mostly caused by time constraints of the students and resulting scheduling conflicts. However, we cannot exclude the possibility of self-selection by high-performing, highly motivated students to finish the course. This could potentially cause a bias towards higher performance during session 3 and 4. To assess the likeliness of this scenario, we compared performance during the first two sessions for students who would finish the course and those who would not. We did not find differences in performance for these groups and thus performance bias caused by self-selection is not likely to have impacted our results.

As this study was performed in an educational setting at the internship stage without proficiency-based criteria, it is not immediately clear what the ramifications are for the operating room, and follow-up translational work is needed. However, studies such as ours show that setting explicit goals followed by summative feedback does impact performance, and this ultimately can contribute to the culture of safety in and out of the operating room.

Students knew whether they were training speed or damage control, which could make it conceivable that performance differences were not caused by the different feedback, but simply because the participant tried to perform faster or with lesser damage. However, in an unpublished pilot study where students were solely instructed to focus on either speed or damage control and feedback did not differ, we did not found differences in performance in the data. Therefore, we expect differences in performance between the two training series in this study to be caused by the different feedback.

### Future research

We are only starting to understand the relations between individual differences and surgical performance. We have begun to study impulsiveness, relevant for damage control [4, 7–12, 46], but other individual differences need to be taken into account as well. Personality includes more aspects than just impulsiveness which need to be investigated. Also, for the spatially challenging aspects of minimally invasive surgery for instance visuospatial ability is a relevant individual difference [47]. Team dynamics in the operating room are likely to be impacted by personality, and ‘Big Fife’ personality traits need to be studies in this context (as has been done in fields such as product design and nuclear powerplant operation [48, 49]). The better we can predict surgical performance based on individual differences, the more efficient and effective our adaptive training systems can be.

Research in this area however would be complex, requiring large datasets to address the relations between the many variables of interest. An approach to speed up this effort might be to use digital simulation training and digital testing for relevant individual differences in a multi-institutional effort to collect the required data. Given the dynamic, complex, and incomplete nature of such data, a machine learning approach based on Bayesian network modeling would be necessary to expedite the analysis of such data [50]. Dynamic, real-time analysis and modeling would open up exciting possibilities for adaptive training, team selection, and case assignment.

## Conclusion

Targeted, selective feedback on selected performance measures can be used to alter training focus and performance. Trainee impulsiveness did not modulate this effect, and selective feedback can be used to design adaptive training systems that mitigate the negative effect of impulsiveness on damage.

## Data Availability

The datasets used and analyzed during the current study are available from the corresponding author on reasonable request.

## Abbreviations

VR: Virtual reality
FLS: Fundamentals of Laparoscopic Surgery
